# Cationic protein 8 plays multiple roles in *Galleria mellonella* immunity

**DOI:** 10.1038/s41598-022-15929-6

**Published:** 2022-07-11

**Authors:** Jakub Kordaczuk, Michał Sułek, Paweł Mak, Agnieszka Zdybicka-Barabas, Justyna Śmiałek, Iwona Wojda

**Affiliations:** 1 Department of Immunobiology, Institute of Biological Sciences, Maria Curie-Sklodowska University, Akademicka 19, 20-033 Lublin, Poland; 2 Department of Analytical Biochemistry, Faculty of Biochemistry, Biophysics and Biotechnology, Jagiellonian University, Kraków, Poland

**Keywords:** Innate immunity, Peptides, Biochemistry, Immunology

## Abstract

*Galleria mellonella* cationic protein 8 (GmCP8) is a hemolymph protein previously identified as an opsonin and an inhibitor of fungal proteases. In this work, we showed its bactericidal activity toward *Pseudomonas entomophila*, *Pseudomonas aeruginosa*, *Bacillus thuringiensis*, *Staphylococcus aureus*, and *Escherichia coli* and against yeast-like fungi *Candida albicans*. The activity against *E. coli* was correlated with bacterial membrane permeabilization. In turn, in the case of *P. entomophila*, *B. thuringiensis,* and *C. albicans*, the atomic force microscopy analysis of the microbial surface showed changes in the topography of cells and changes in their nanomechanical properties. GmCP8 also showed the inhibitory activity toward the serine protease trypsin and the metalloproteinase thermolysin. The expression of the gene encoding the GmCP8 protein did not increase either in the gut or in the fat body of *G. mellonella* after oral infection with *P. entomophila*. Similarly, the amount of GmCP8 in the hemolymph of *G. mellonella* did not change in immune-challenged insects. However, when GmCP8 was injected into the *G. mellonella* hemocel, a change in the survival curve was observed in the infected larvae. Our results shed new light on the function of GmCP8 protein in insect immunity, indicating its role in humoral defence mechanisms.

## Introduction

*Galleria mellonella* is one of the most frequently applied insect models in studies of innate immunity, antimicrobial peptides, host–pathogen interactions and in tests of human pathogens and antimicrobial drugs^1,2^. The genome of *G. mellonella* was sequenced in 2018. It contains over 14,000 genes, encoding, e.g. plenty of proteins and peptides involved in immune response^3^. Among them, there are also proteins participating in the first step of immune reaction, namely recognition of infection. Pattern recognition receptors (PRRs) bind to so-called pathogen-associated molecular patterns (PAMPs), for example lipopolysaccharide (LPS), β-glucan, peptidoglycan (PG), and lipoteichoic acids (LTA). PRRs are represented by peptidoglycan recognizing proteins (PGRPs), which bind and sometimes also digest bacterial peptidoglycan, Gram-negative binding proteins (GNBP), which recognize and bind fungal β-glucan. Apolipophorin-III (apoLp-III)^4,5^ and hemolin^6^ are the other proteins serving in *G. mellonella* and other Lepidoptera as PRR. They bind to LPS, LTA, and fungal β -1,3 glucan^4–6^. Down Syndrom Cell Adhesion Molecules (Dscam) are characterized by diversity because some exons of their gene contain alternative forms encoding the part of the protein that binds pathogens. Thanks to alternative splicing, insects are able to produce thousands of Dscam receptors binding pathogens with different specificity. In the *G. mellonella* genome, components of Toll and Imd pathways, as well as components of JAK/STAT pathways were found^3^. Analysis of the transcriptome also indicated that *G. mellonella* produces four chicken-type and one i-type (*invertebrate type*) lysozymes^7^. These enzymes are muramidases digesting bacterial peptidoglycan and act against Gram-positive bacteria, some Gram-negative bacteria, and some fungi^8,9^. There are also reports indicating non-enzymatic antimicrobial activity of *G. mellonella* lysozyme, which is known to act synergistically with antimicrobial peptides^10^.

About 20 members of antimicrobial peptides (AMPs) have been isolated from *G. mellonella* hemolymph so far^11–16^. Among defensin-like peptides, whose structure is stabilized by disulfide bridges, there are gallerimycin (6.4 kDa), galiomycin (4.7 kDa), and a so-called defensin-like peptide (4.9 kDa) with activity directed against filamentous fungi^14,17–19^. The group of linear peptides is represented by cecropins (Cec). CecA with approx. 4.2-kDa molecular weight was identified in the hemolymph of immunized larvae has great homology to cecropin A from *Hyalophora cecropia*^20^. 4.3-kDa cecropin D shows homology to cecropins of *Manduca sexta* and *Bombyx mori*^13,21^. Moricins, whose presence is restricted to Lepidoptera, owe their name to *B. mori*. They are α-helical peptides with antibacterial and antifungal properties^22^. Seven moricins encoded by eight genes and five gloverin transcripts were found in *G. mellonella*^7,16,22^. Initially, gloverin was found in *H. gloveri*^23^. These peptides belong to the group of AMPs, which are rich in glycine but have cysteine residues in the molecule^17,24^. In turn, proline-rich AMPs were named proline peptides 1 and 2 (Pro-1 and Pro-2). They have molecular weight of 4.3 and 4.9 kDa, respectively. As in the case of gloverins, their activity is associated with an increase in the permeability of the microbial membrane^13,14,16,25^. Proline-rich peptides have a common transcript with the heliocin-like peptide homologous to the *Heliothis virescens* peptide and anionic peptide-1 (AP1)^16^. Another anionic peptide found in *G. mellonella* is anionic peptide-2 (AP2), which is active against Gram-positive bacteria and fungi. In contrast to most AMPs, anionic peptides are negatively charged and bind to positively charged regions of the bacterial membrane. The structure of 4.3-kDa AP1 is similar to that of the lebocin precursor from *B. mori*^14,26^, while AP2 (6.98 kDa) has no similarity to any known AMPs. Inducible metalloproteinase inhibitor (IMPI) with molecular weight of approx. 8.4 kDa was the first discovered specific inhibitor of metalloproteinases^15,27^. It contains five disulfide bridges and inhibits the activity of metalloproteinases having zinc in their active centre, e.g. these from the thermolysin group^28^. Despite the presence of a TIL domain-trypsin inhibitors of serine proteinases-, it does not inhibit the activity of these enzymes^29^. Proteases are secreted by bacteria as virulence factors and digest host immune proteins and peptides.

Cationic protein 8 from *G. mellonella* (GmCP8) was first isolated from larval hemolymph by Fröbius and co-workers^12^. The authors reported an increase in the protease inhibitory activity of *G. mellonella'* hemolymph after zymosan injection and named the isolated enzymes ISPI-1-3 (inducible serine protease inhibitors 1-3); however, they did not estimate the inducibility of individual proteins. ISPI-1 (GmCP8) showed no sequence similarity to any known inhibitor of proteases. It inhibited the activity of bovine pancreatic trypsin and Pr2 protease from *Metarhizium anisopliae*^12^*.* Its deduced aminoacid sequence was submitted as XP_026758048.1 (fungal protease inhibitor, FPI). The same protein was further identified as one of three proteins detached from the cell wall of heat-killed *C. albicans* and named FBP1 (Fungal Binding Protein 1). Other two proteins detached from the fungal cell wall, i.e. FBP2 and FBP3, were identified as lysozyme and defensin-like peptide, respectively^19^. Six years later, Kim et al.^30^ isolated a 9 kDa protein with high sequence homology to these mentioned above and to the cationic protein-8 from *Manduca sexta* and named it GmCP8 by analogy. This protein (deposited as ADI87454.1) was shown to bind to LPS, LTA, and β-1,3-glucan, and this binding was necessary for phagocytosis of *E. coli*, *M. luteus,* and *C. albicans*, respectively. The protein was detected in the fat body and cell-free hemolymph (but not in hemocytes), while the transcripts were found in the fat body and integument; they remained at the same level after injection of *E. coli*. It appeared that ISPI-1 (FPI-1) and GmCP8 are the same protein, as can be concluded from the comparison of their deposited sequences in the NCBI database (Supplementary Fig. S1 online). GmCP8 exists in two forms, differing in the presence/absence of C-terminal glycine, and both consist of 12 cysteines in the molecule. Proteins with high similarity were found in other organisms, e.g. *M. sexta*, *B. mori*, *B. mandarine,* and *Antheraea mylitta*^31,32^. AmCP8 (AmFPI-1) inhibited protease from *Aspergillus oryzae* and showed 46% identity to GmCP8 (GmFPI-1). AmFPI-1 also contains 12 cysteines and is likely to form six disulfide bridges, which may account for high thermal stability of the protein^31^. On the other hand its 9.1 kDa orthologue from *M. sexta*, called a small cationic protein, did not inhibit the activities of seven proteases tested but stimulated phenoloxidase (PO) activation in hemolymph^32^. All orthologues of GmCP8 were shown to be expressed in the fat body, to be secreted to the hemolymph, and to contain 12 cysteines in the molecule^12,30,32^.

In search for defence molecules in the low molecular weight fraction of *G. mellonella* hemolymph after infection with the entomopathogenic bacteria *Pseudomonas entomophila*, we paid attention to a > 9 kDa protein which was abundant in one of the HPLC fractions. We identified it as GmCP8, purified it, and tested its defence properties. In the present paper, we show that this protein functions as an inhibitor of both trypsin and thermolysin and has activity against several microorganisms. This indicates that the protein called GmCP8 plays a significant role in the humoral aspects of *Galleria* immunity.

## Results

### Purification and identification of GmCP8 protein from *G. mellonella* hemolymph

**Figure 1:**
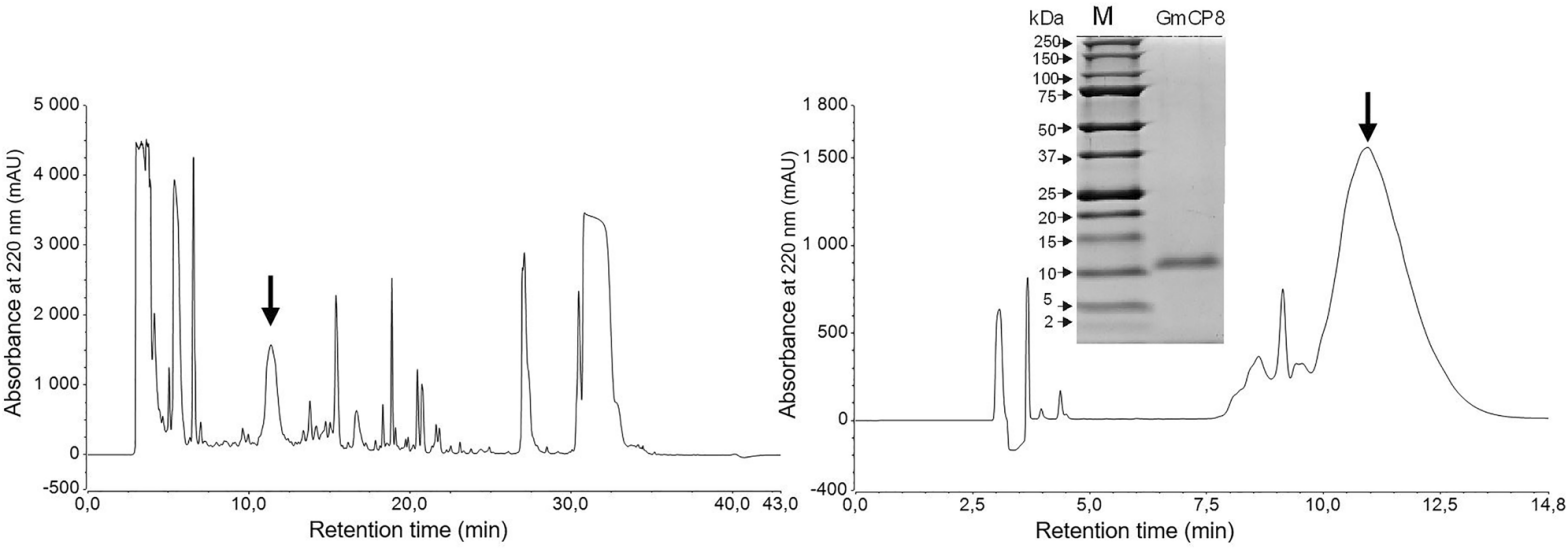
RP-HPLC chromatograms from two successive steps of GmCP8 purification. The arrows denote collected fractions, while the insert shows the electrophoretic image of the purified protein. The image of purified GmCP8 was made with the use of ChemiDoc MP Imaging System using Image Lab version 4.1 software (Biorad). Non cropped GmCP8 picture is presented in Supplementary Fig. S2.

GmCP8 was purified by RP-HPLC from the hemolymph of *G. mellonella* larvae immunized with a mixture of *E. coli* and *M. luteus*. The obtained GmCP8 preparation was homogenous in SDS/PAGE, which showed a single band localized slightly above the 10-kDa marker (Fig. 1). The N-terminus of the protein was sequenced by Edman degradation and identified the protein as GmCP8 (*G. mellonella* cationic protein 8), i.e. a multiligand recognition protein functioning as an opsonin and fungal protease inhibitor FPI1, as described in the Introduction^12,19,30^. The mass spectrometry analyses confirmed the identity of the protein and revealed the presence of non-modified polypeptide chains with molecular weight 9168 Da and 9227 Da, differing in only the presence/absence of the last C-terminal glycine^30^ (Supplementary Fig. S3 online).

### GmCP8 acts as an antimicrobial peptide

**Figure 2:**
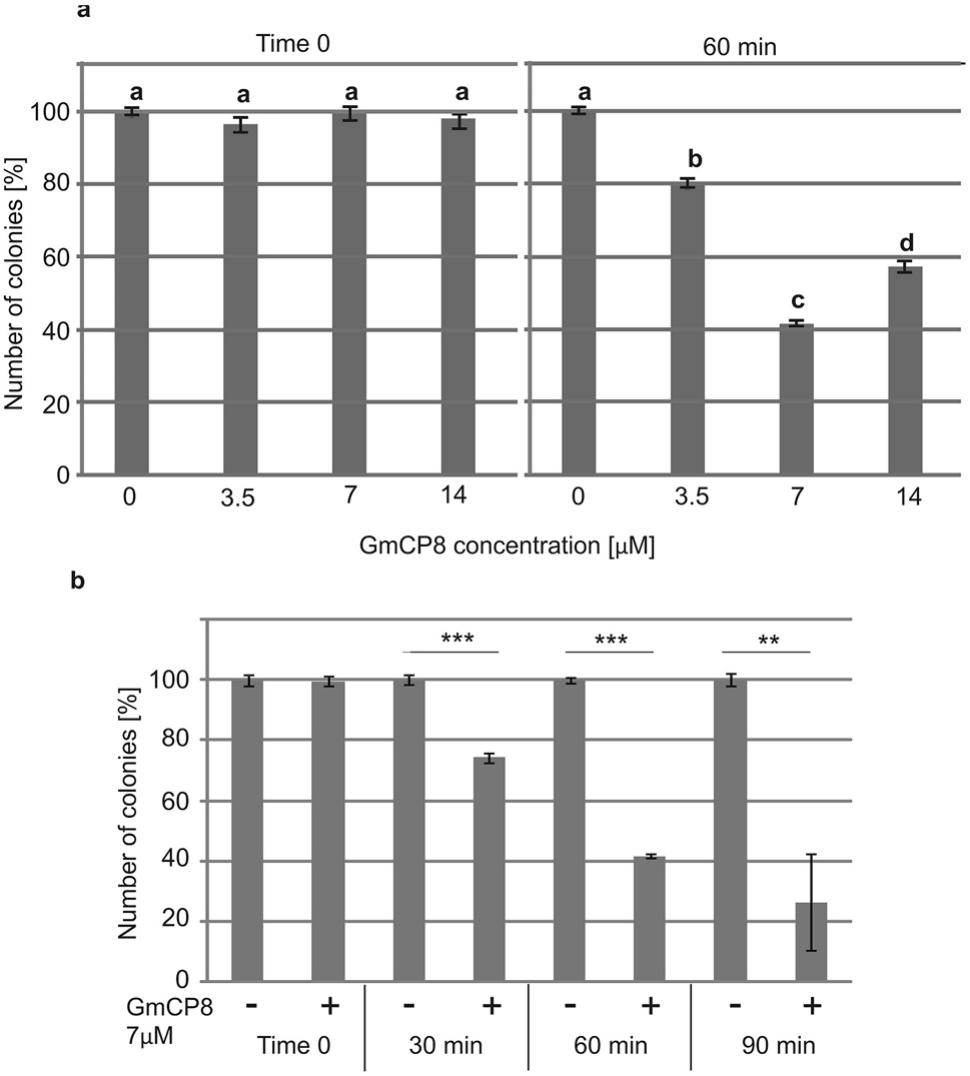
Antimicrobial activity of the GmCP8 peptide against *P. entomophila*. (**a**) GmCP8 at different final concentrations was mixed with *P. entomophila* as indicated in the “Materials and methods”. (**b**) GmCP8 at a concentration of 7 µM was incubated with *P. entomophila* for the indicated time. In the control, an equal amount of water was added instead of GmCP8. The samples were plated directly (time 0) and after the indicated time of incubation. The results are shown as a percentage of CFU grown after incubation with the GmCP8 peptide in relation to the average number of CFU without addition of the peptide at the respective time points. The experiment was performed three times, and data from 2 dilutions were taken for calculations each time. Mean values ± SD are shown. In (**a**)—data with different letters show significant difference (One-way ANOVA, Tukey’s test, p < 0.05). In (**b**)—significant differences are indicated * p < 0.05, ** p < 0.01, *** p < 0.001 (Student t-test).

**Figure 3:**
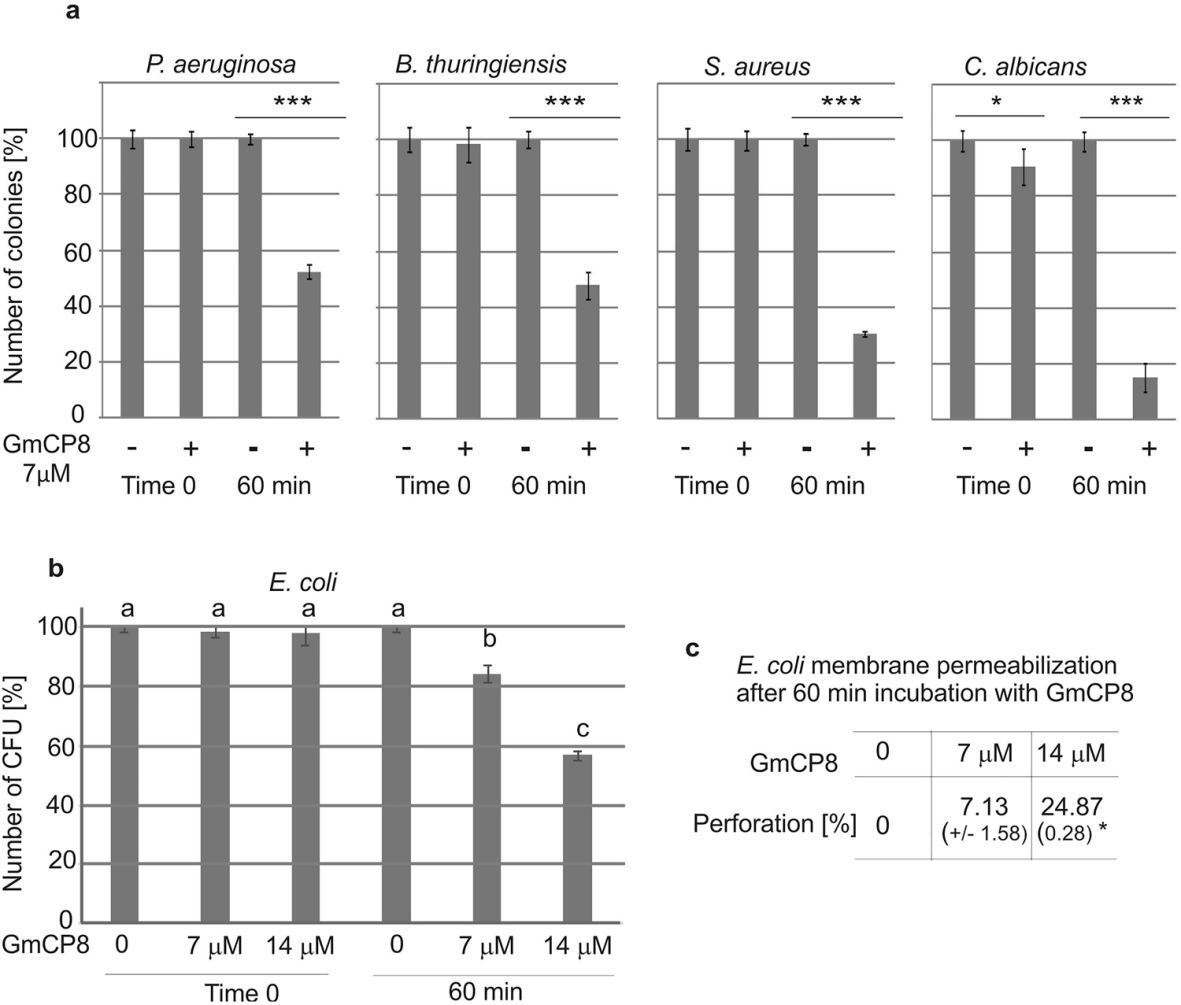
Antimicrobial activity of the GmCP8 peptide against different microorganisms. (**a**) GmCP8 (7 µM) was mixed with Gram-positive bacteria (*B. thuringiensis, S. aureus*), Gram-negative bacteria (*P. aeruginosa*), and fungi *C. albicans* as indicated in the “Materials and methods”. In the control, an equal amount of water was added instead of GmCP8. The samples were plated directly (time 0) and after 60-min incubation. The results are shown as a percentage of CFU grown after incubation with the GmCP8 peptide in relation to the average number of CFU without addition of the peptide. Mean values ± SD are shown. The experiments were performed three times and data from 2 dilutions were taken for calculation each time. Significant differences are indicated *p < 0.05, **p < 0.01, ***p < 0.001 (Student’s t-test). (**b**) Activity of GmCP8 against *E. coli* JM83 was determined as described above but two GmCP8 concentrations were used: 7 µM and 14 µM. Mean values ± SD are shown; different letters show statistical difference (p < 0.05, ANOVA, Tukey’s test). (**c**) Membrane permeabilization determined by measuring β-galactosidase leakage into the medium after treatment with GmCP8. The perforation level of the dead bacteria was assumed as 100% as described in the “Materials and methods”. The values represent means from 3 permeabilization assays ± SD. Statistical significance between the results obtained with two GmCP8 concentrations is shown (*p < 0.05, Welch's t-test).

The GmCP8 peptide was checked for its antimicrobial activity using the colony counting method. We tested its activity against *P. entomophila* at three concentrations (3.5 µM, 7 µM, and 14 µM). The peptide appeared to act against *P. entomophila*, with the highest activity observed at 7 µM (Fig. 2a). The 60-min incubation was sufficient for detecting its reproducible activity (Fig. 2b). Additionally, the peptide was active against other entomopathogenic bacteria *B. thuringiensis*, human opportunistic pathogens *P. aeruginosa* and *S. aureus*, and fungi *C. albicans* (Fig. 3a)*.* The concentration of 7 µM of GmCP8 against *E. coli* reduced the number of CFU by only 20%; therefore, we also checked the higher concentration of 14 µM, which resulted in 40% reduction of CFU (Fig. 3b). The concentration-dependent antibacterial activity directed against *E. coli* correlated with the activity of β-galactosidase released from bacteria, reflecting permeabilization of their membrane, which increased from 7% at 7 µM to almost 25% at 14 µM (Fig. 3c).

### GmCP8 causes changes on the surface of microorganisms

**Figure 4:**
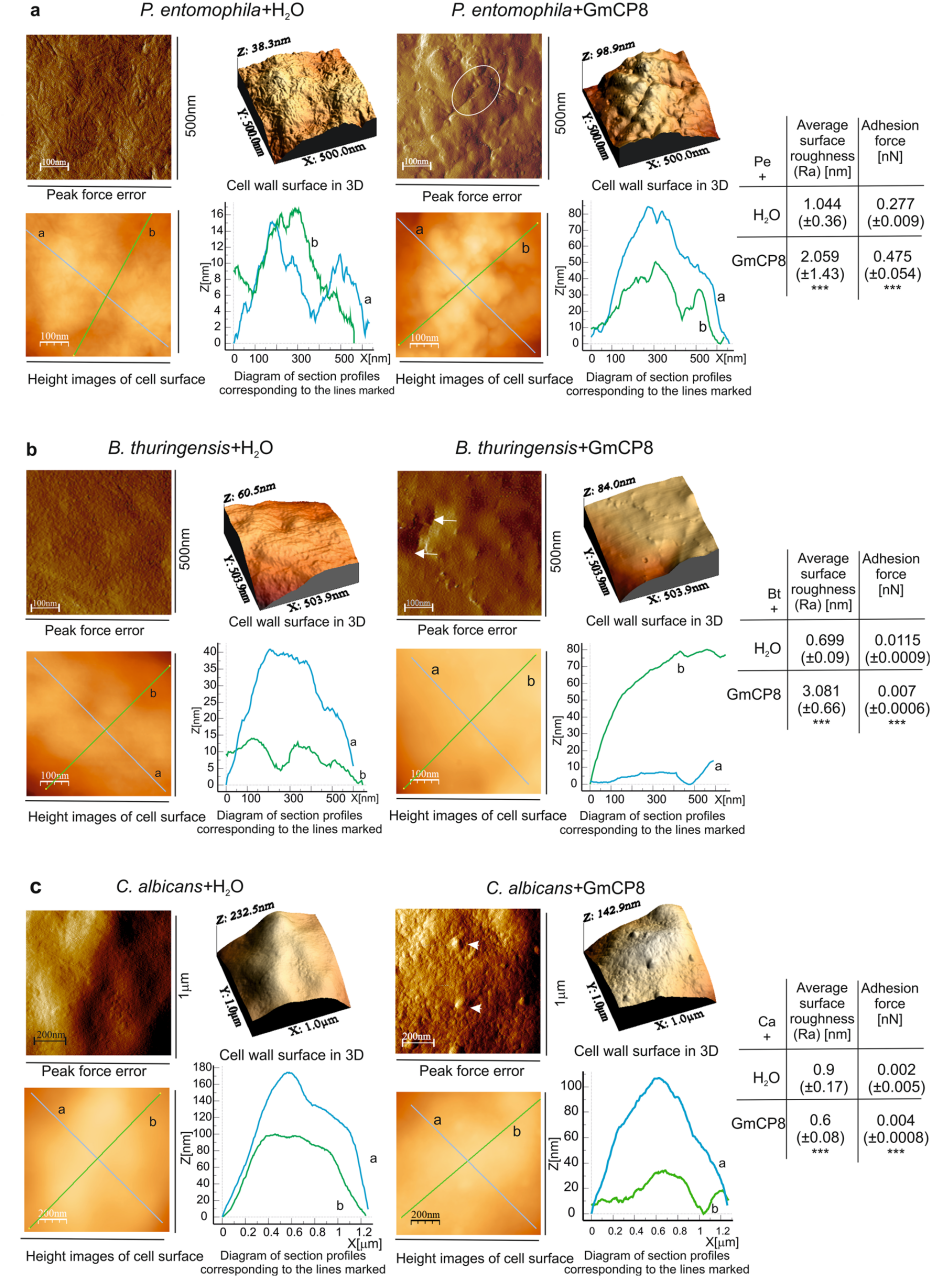
Effect of the GmCP8 protein on (**a**) *P. entomophila,* (**b**) *B. thuringiensis*, and (**c**) *C. albicans* cell surface topography. The microorganisms in the logarithmic growth phase were incubated without (water) or with the GmCP8 protein for 1 h. Then, the cells were imaged by AFM. The peak-force-error, three-dimensional (upper panels in each part), and height images are presented (area of 500 nm × 500 nm for *Pe*, 1 µm × 1 µm for *Bt* and *Ca*) as well as cell surface change profiles measured along lines a and b marked in the height image (lower panels in each part). The alterations in the cell surface are marked by: (**a**) white ellipse indicating grooves; (**b**) white arrows show holes; **c** white arrowheads indicate lumps. The tables show biophysical parameters of the cell surface, such as roughness and adhesion force values. Mean values ± SD are shown, n = 114. Significant differences are indicated *p < 0.05, **p < 0.01, ***p < 0.001 (Mann–Whitney U test). The images were taken using Nanoscope Analysis vl. 40 (Veeco), the section profiles and three-dimensional (3D) images of the cells were generated using WSxW 5.0 software (Nanotec, Spain)^66^.

With the use of atomic force microscopy (AFM), we were able to analyze changes in the microbial surface structure and biophysical parameters after incubation with the GmCP8 peptide. We tested the surface of *P. entomophila* as a representative of Gram-negative bacteria, *B. thuringiensis* (Gram-positive), and fungi *C. albicans* (Fig. 4). Peak force error images show the cell surface topography and visible differences in the surface where *P. entomophila* lost their typical morphology and regular nut-like structure. The three-dimensional image (3D) of the bacterial surface profile revealed that the distance between the highest and the lowest point of the surface was greater in cells incubated with the peptide (as indicated on the Z axe: 98.9 nm) than in the control cells (38.3 nm), as the bacterial surface in the experimental variant was rougher (Fig. 4a, top panel). The alterations in the cell surface of *P. entomophila* incubated with the peptide were evidenced by the presence of long grooves. The cell surface profiles (Fig. 4a, bottom panel) revealed that the grooves were deeper and had various diameters (approx. 20–30 nm deep and 100–150 nm in diameter). The topography alterations were accompanied by changes in biophysical parameters (Fig. 4a, table), such as the surface roughness value, which increased significantly (approx. twice) to 2.059 (± 1.43) nm. Additionally, adhesion forces increased almost twice after the incubation with the peptide in comparison with the control cells, showing changes in the biophysical properties of the cell surface (Fig. 4a).

The changes were also observed on the surface of *B. thuringiensis* cells after the exposure to GmCP8 (Fig. 4b). The regular surface structure was lost: it was covered by granules and irregular deep hollows appeared (indicated by arrows, Fig. 4b, top panel). Measurements along lines "a" and "b" on the height image showed 14–17 nm deep and up to 150 nm wide indentations (Fig. 4b, bottom panel). The morphological changes induced by the peptide were accompanied by changes in cell surface properties, such as a statistically significant increase in the value of roughness to 3.081 (± 0.66) nm and a decrease in adhesion forces (by approx. 1.5-fold to 0.007 nN) compared to the control (Fig. 4b, table).

As in the case of the bacteria, the *C. albicans* surface also lost its regular pattern with appearance of irregular lumps (indicated by arrowheads, Fig. 4c, top panel). The surface of the cells treated with GmCP8 was more granular/lumpy and heterogeneous than the control; however, the surface profile was lower than in the control cells (Fig. 4c, bottom panel), and the damage to the cells resulted in a decrease in the Ra (roughness average) value by approx. 30% to 0.6 (± 0.08) nm, but the adhesion forces doubled (Fig. 4c, table). These biophysical parameters differ from those ones described for the bacterial cells.

### Inhibition of proteases by GmCP8

**Figure 5:**
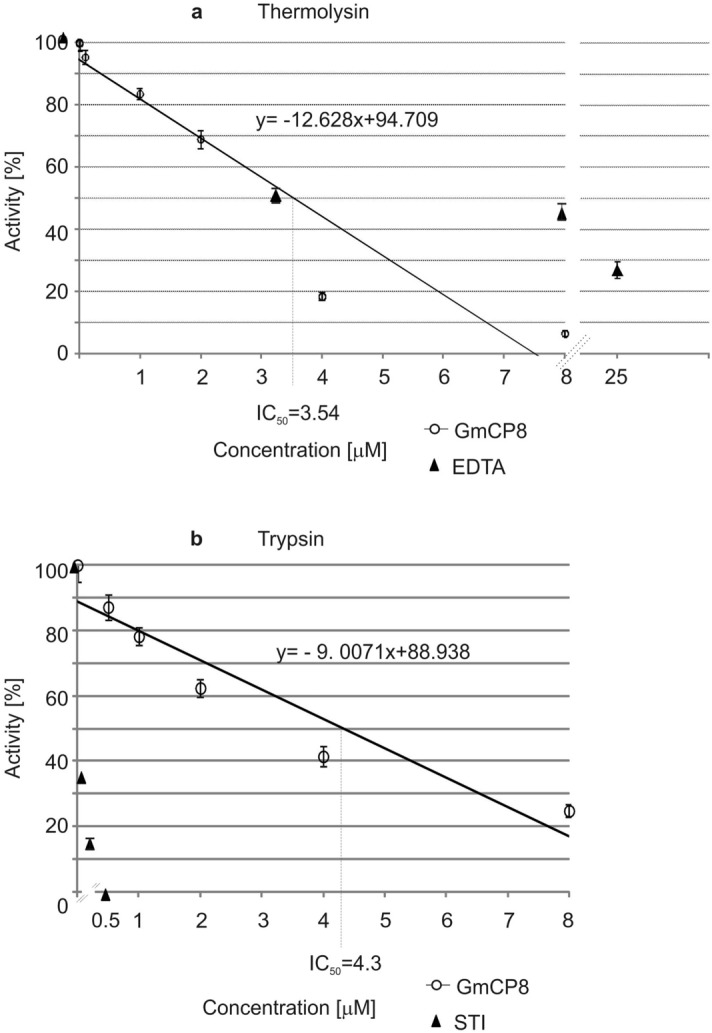
Effect of different concentrations of GmCP8 on the activity of: (**a**) thermolysin; (**b**) trypsin. The results show the mean values from three assays ± SD. EDTA (**a**) and STI (**b**) were used as positive controls and are shown as points. In (**b**), the values for STI between 0 and 0.5 µM are 0.05 and 0.1, which is not shown on the X axis to keep the graph readable. The results obtained for GmCP8 are shown together with trend lines. The IC_50_ of GmCP8 were calculated from the formulas of the respective lines. The control group (100% relative activity) contained water instead of the peptide.

The GmCP8 peptide inhibited the activity of two proteases, namely the serine protease trypsin and the zinc proteinase thermolysin. With the use of the different concentrations of the GmCP8 protein against these enzymes, we established that the half-maximal inhibitory concentration (IC_50_) of GmCP8 is 3.54 µM toward thermolysin and 4.3 µM toward trypsin (Fig. 5).

### Gene expression and amount of GmCP8 in infected larvae

**Figure 6:**
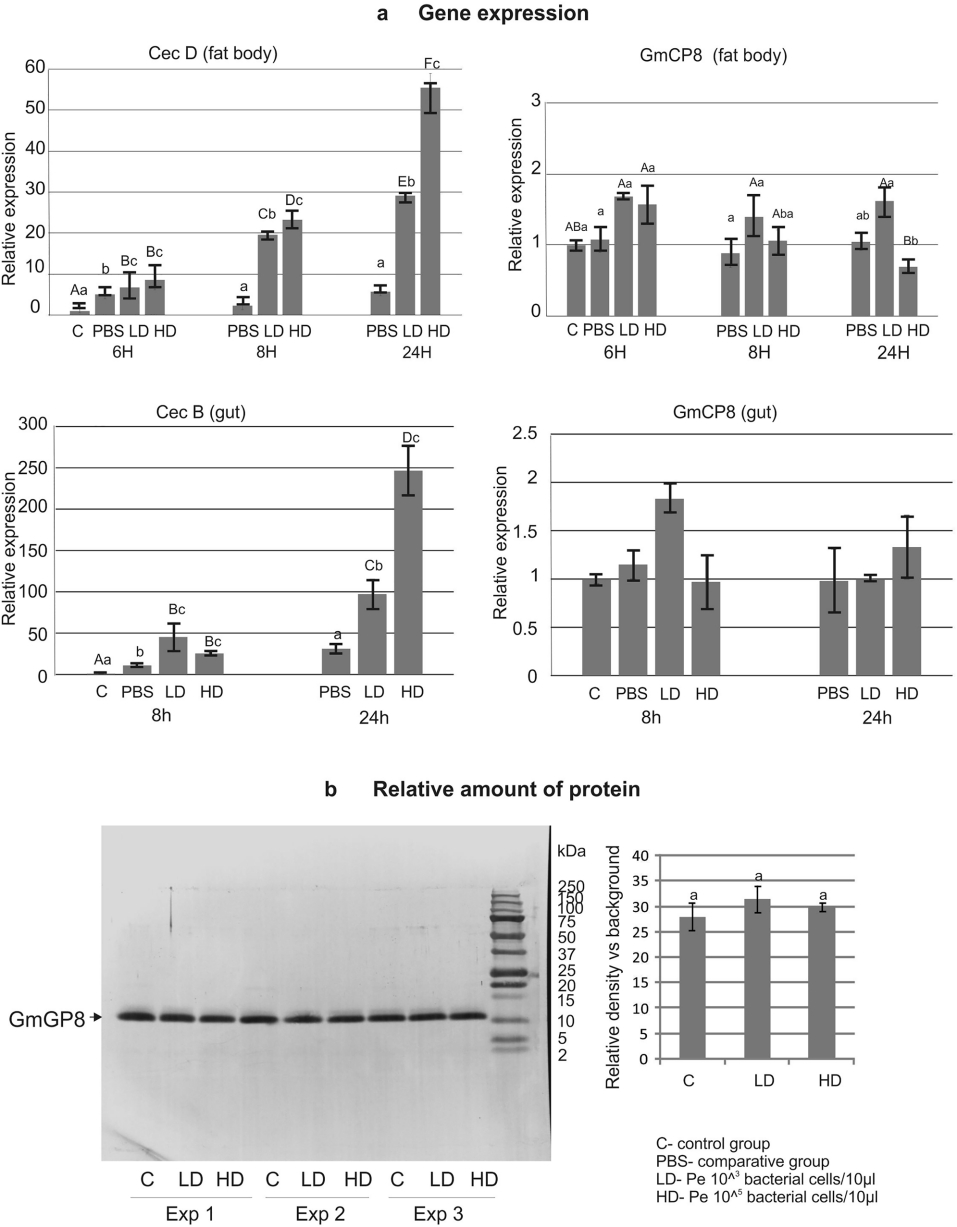
(**a**) Relative expression of genes encoding cecropin and GmCP8 in the fat body and gut after oral infection with the higher (HD, 10^5^ CFU in 10 µl) and lower (LD, 10^3^ CFU in 10 µl) dose of *P. entomophila*. Mean values ± SD from three assays are shown. Significant differences are indicated at p < 0.05 (Kruskal–Wallis One-Way ANOVA, Tukey’s-post-hoc tests); small letters—differences between different groups at the same time point, big letters—differences between different time points in the same group; (**b**) Comparative quantitative analysis of the GmCP8 protein present in *G. mellonella* methanol extracts separated by HPLC. Left: stained membrane with separated proteins transferred after protein electrophoresis of the GmCP8-containing fraction, the control (water administration), and infection with LD and HD of *P. entomophila* larvae from 3 experiments. The protein was identified by N-terminal sequencing by Edman degradation. Right: relative density band representing the GmCP8 protein; mean ± SD from 3 experiments. No significant differences were found in samples from the infected larvae and the control (p < 0.05, ANOVA).

As shown in Fig. 6a, the expression of the GmCP8 gene was not induced by the oral infection of the larvae with *P. entomophila,* while the expression of the immune marker gene of cecropin D was up to 60 and over 270 times induced in the fat body and in the gut, respectively. We also analyzed the amount of HPLC-separated protein after the oral infection by measurement of the peak area (Supplementary Fig. S4 online) and by electrophoresis of the same fraction (Fig. 6b). No significant differences were found in the amount of GmCP8 in *G. mellonella* hemolymph after the oral infection. Additionally, the amount of the protein in the hemolymph extracts did not change when the bacteria were administered by intrahemocelic injection (Supplementary Fig. S4—quantitative HPLC and Supplementary Fig. S6—electrophoretic analysis, online).

### Effect of GmCP8 on the survival curve of infected *G. mellonella* larvae

**Figure 7:**
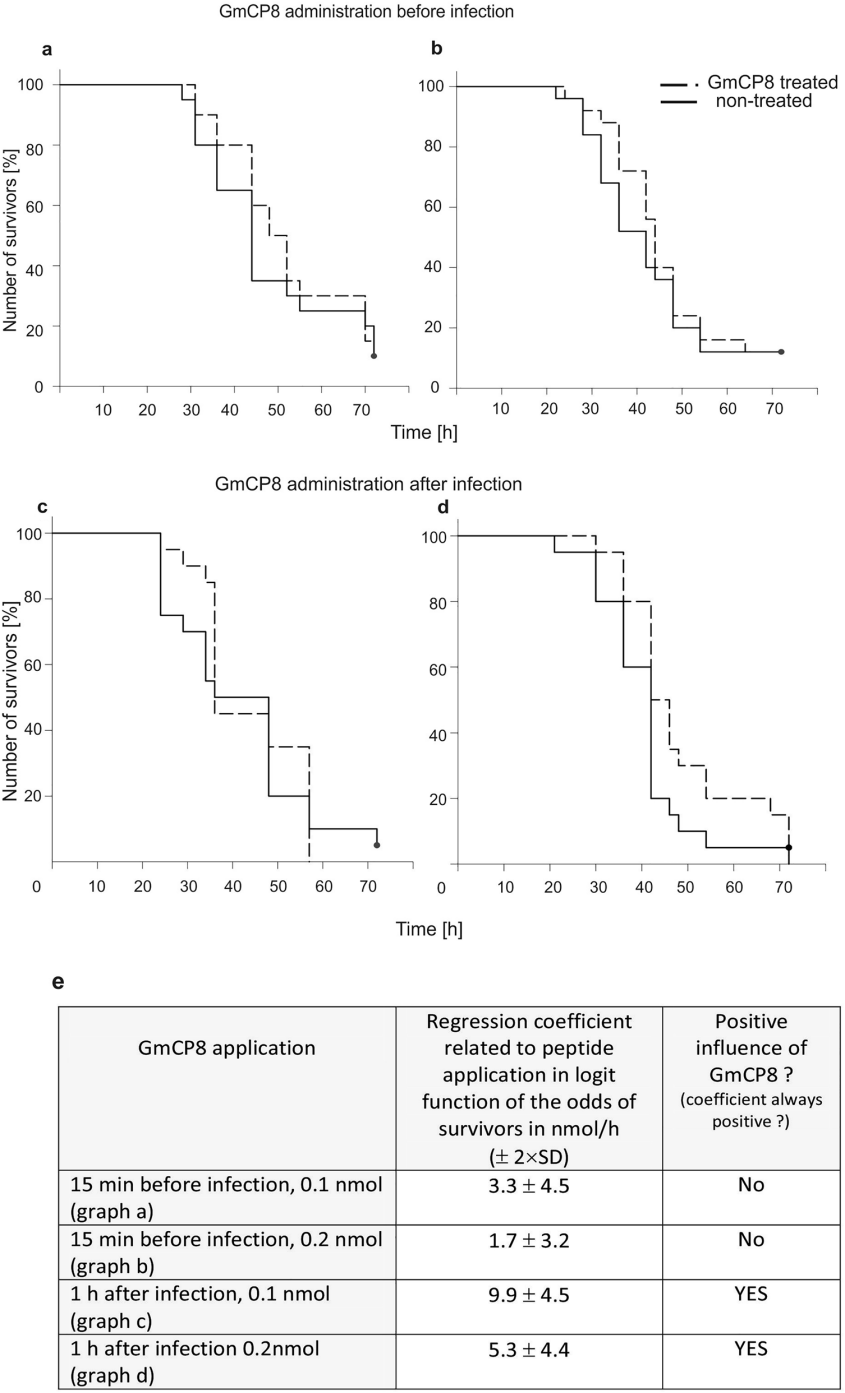
Kaplan-Maier survival curves of *G. mellonella* larvae after intrahemocelic administration of 0.1 nmol (**a** and **c**) and 0.2 nmol (**b** and **d**) of GmCP8 (treated larvae) and water (non-treated larvae) 15 min before (**a** and **b**) and 1 h after (**c** and **d**) injection of *P. entomophila*. In (**b**)—the groups comprised 25 larvae each; in: (**a**,**c**,**d**) the groups comprised 20 larvae each. (**e**) result of the binomial regression analysis of each experiment (**a**–**d**) with respect to the effect of GmCP8 in comparison to the application of water. Regression coefficients related to the peptide application in the logit function of the odds of survivors is positive (above 0) in each case; taking into consideration the standard deviation (± 2 SD), the positive effect of GmCP8 on the infected larvae was statistically confirmed, when GmCP8 was applied 1 h after the administration of bacteria.

Further, we checked the survival curves of infected larvae after the injection of GmCP8. We chose two time points of peptide application: 15 min before the bacterial injection, which gave the highest possibility that the peptide is already distributed in the hemolymph but not yet digested by the insect's proteases, and one hour after the bacterial injection, i.e. the time-point that the infection is recognised and the first transcripts of immune-induced genes are detectable^33^ but the antimicrobial activity of the hemolymph is not yet detected in vitro (our observation). When the GmCP8 peptide in the amount of 0.1 and 0.2 nmole per larvae was injected into *G. mellonella* 15 min before and one hour after the infection with *P. entomophila*, we noticed not a spectacular but consistent slight shift in the course of Kaplan–Meier curves in the group treated with GmCP8 (Fig. 7a–d). The binomial regression analysis showed statistically significant positive effect of GmCP8 on the infected *G. mellonella* larvae in the case where GmCP8 was applied 1 h after the bacterial injection, although the values of the coefficient were not high, which indicates a rather slight effect (Fig. 7e).

## Discussion

*Galleria mellonella* is a rich source of bioactive molecules with microbicidal or immune properties^2^. We report here characterization of *G. mellonella* hemolymph protein with molecular weight of approx. 10 kDa, corresponding to the LOC113517546 gene, encoding a protein previously reported as fungal protease inhibitor ISPI-1 and GmCP8^12,19,30^. Its aminoacid sequence shows similarity to the 10.4-kDa protein purified from the Indian tasar silkworm *Antheraea mylitta*, which was reported to inhibit fungal proteases^31^ and to the 8-kDa cationic protein (CP) from *M. sexta*^30,32^. The GmCP8 protein was previously characterized as a multiligand recognition protein, which functions as an opsonin participating in cellular reactions^30^. Despite the different names reflecting similarities to other proteins, the GmCP8 protein is not fully characterized and its role in *G. mellonella* immunity is only partially known^30^. The GmCP8 protein was present in significant amounts in the methanol extract of *G. mellonella* hemolymph. Its amount did not change after infection of *G. mellonella* larvae with *P. entomophila* either orally or by intrahemocelic injection. We showed that the GmCP8 protein has antimicrobial activity, since it was active against Gram-positive bacteria (*B. thuringiensis*), Gram-negative bacteria (*P. entomophila*, *P. aeruginosa*, *E. coli*), and fungi *C. albicans*. The highest activity against *P. entomophila* was observed at 7 µM. However, in the case of *E. coli* carrying β-galactosidase-encoding plasmid pCH110, the antibacterial and β-galactosidase activities were higher at 14 µM of GmCP8, which was correlated with a higher level of membrane permeabilization.

The activity against different microorganisms was correlated with changes on their surface detected with the use of AFM. The surface of *P. entomophila* lost its "nut-like" structure; it became rougher and had long and deeper groves. Additionally, their adherence increased significantly, indicating that GmCP8 changed the nanomechanical properties of the cell surface. The loss of regular structure and the increase in roughness and adhesion were observed in *B. thuringiensis* exposed to GmCP8, while C*. albicans* surface became more granular and heterogeneous. Other studies showed that, upon binding to the cells of Gram-negative bacteria *E. coli* JM83, cecropin D purified from *G. mellonella* hemolymph changed their characteristic topography and nanomechanical properties. The changes imaged by AFM depended on the peptide concentration and the time of interaction with the bacterial cells^34^.The results obtained for bacteria *E. coli* JM83 upon contact with *G. mellonella* apoLp-III revealed that the cell surface became rougher in comparison to the control cells and these changes were accompanied by changes in the biophysical properties. Moreover, altered surface texture of *E. coli* cells was also detected after treatment with *G. mellonella* lysozyme alone and together with apoLp-III of *G. mellonella* or after incubation with synthetic cecropin A from *G. mellonella*^35,36^. Also, incubation of *E. coli* JM83 cells with *Lucilia sericata* peptides, namely stomoxyn or the proline-rich peptide PRP2, alone and in combination resulted in changes in cell topography and increased roughness and adhesion forces, compared to controls^37^. The use of AFM helped to demonstrate significant changes in the structure of the cell surface of such bacteria as *Bacillus circulans, Micrococcus luteus, Klebsiella pneumoniae, Salmonella typhimurium* and *Legionella pneumophila* after treatment with *G. mellonella* apoLp-III, which also has direct antimicrobial properties. ApoLp-III disturbed the proper structure of the bacterial cell surface and caused an increase in RMS roughness, compared to control^38,39^. AFM imaging demonstrated evident alterations in the fungal surface of *C. albicans, Zygosaccharomyces marxianus,* and *Fusarium oxysporum* after incubation with apoLp-III^40^. The potential of antifungal activity against *C. albicans* was observed as alterations in the surface nanomechanical parameters of cells after interaction with *G. mellonella* anionic peptide 2 (AP2) or *G. mellonella* lysozyme^8,41^. Atomic force microscopy imaging suggests that antimicrobial peptides and proteins exert their action by targeting the microbial surface and disrupting the membrane integrity. The mechanism dependent on electrostatic attractions between the polycationic peptides and the anionic microbial surface, probably followed by insertion into the cytoplasmic membrane and permeabilization thereof, is supported by the biophysical results.

The antimicrobial properties of GmCP8 place this protein among other antimicrobial peptides that may be applied in vitro to prevent undesirable microbial growth on the surface of biomedical devices, dentures, and implants, on food packaging, and in food and cosmetics preservation^42,43^. The binding of GmCP8 to the bacterial surface is in agreement with the finding that GmCP8 can bind to LPS, lipoteichoic acids, and β-glucans^30^. The cited authors also reported that GmCP8, when bound to pathogens, is internalized into hemocytes together with opsonized microorganisms^30^. Taking into consideration the antimicrobial properties of GmCP8 shown in the present study, it is likely that it can also participate in bacterial killing inside hemocytes. Since the GmCP8 protein has been shown to be an opsonin^20^, to have bactericidal properties, and to be present in the hemolymph of naive larvae and its expression is not immune-stimulated, we may conclude that GmCP8 is a molecule of the first line of *G. mellonella* defence. Also Kim and co-workers did not detect increased expression of GmCP8 after application of non-pathogenic bacteria *E. coli*^20^. Despite the constant presence of the GmCP8 protein in *G. mellonella* hemolymph, this tissue collected from non-challenged larvae does not show significant activity against Gram-negative bacteria when tested with the radial-diffusion method^33,44^. However, this assay is less sensitive than incubation of bacteria in liquid media with an antibacterial compound (our observations). It is also likely that the antimicrobial activity of the GmCP8 peptide is regulated by other hemolymph compounds, as it is well known that insect proteins and peptides co-operate with each other and have an impact on their mutual activity^18,35,45,46^.

There are more proteins with antimicrobial activity in the hemolymph of naive *G. mellonella*. Such an example is apoLp-III, whose direct antimicrobial properties have been proven and whose amount even decreases in the serum of infected *G. mellonella*. ApoLp-III also binds to pathogen-associated molecular patterns (PAMPs) and serves as an opsonin. It also enhances the activity of antimicrobial molecules^46–48^. In addition to its immune functions, apoLp-III serves as a storage protein. It is able to bind and transport lipids to flight muscles supplying them with an energy source^49^. The other examples of defence molecules present in the hemolymph of non-challenged larvae are lysozyme and AP2^47,48^.

Proteases secreted by pathogens play an important role in pathogenicity. They contribute to degradation of host tissues to allow the pathogen to enter the insect's body and degrade host immune-relevant proteins and peptides. Inhibitors of these enzymes are important defence effectors. They include insect inhibitor of metalloproteinases (IMPI), and a number of serine proteinase inhibitors from the Kunitz, Kazal, and serpin families^12,50^. Since GmCP8 shows similarity to the inhibitor of fungal proteases in *Antheraea mylitta* and to ISPI-1, we checked its effect on two proteases: trypsin and thermolysin. We found that GmCP8 inhibited both thermolysin and trypsin activity. In turn, the CP8 protein of *M. sexta*, unlike AmFBP-1, did not inhibit the activity of seven serine proteases^12,31,51^. Thermolysin obtained from *Bacillus thermoproteolyticus* is a zinc-metalloproteinase with similarity to other metalloproteinases secreted by pathogenic organisms as virulence factors^52^. For example, InhA and InhB proteases produced by *B. thuringiensis* degrade insect host immune-related polypeptides allowing bacteria to develop in the body of the infected host^53–55^. The *P. entomophila* genome encodes *aprA*^*c*^ alkaline metalloproteinase and four serine proteases PSEEN^56^. In turn, thermolysin-mediated digestion of hemolymph proteins/peptides generates so-called protfrags, which stimulate induction of host defence mechanisms, e.g. the synthesis of antimicrobial peptides and IMPI (insect metalloproteinase inhibitor)^15,52,54,57^. IMPI was found as the first specific peptide inhibitor of metalloproteinases and its expression is stimulated after immunization. Here, we have shown that the GmCP8 protein may also inhibit the activity of zinc-metalloproteinase; however, unlike IMPI, GmCP8 is constantly present in *G. mellonella* hemolymph. Inhibitors of proteinases are good examples of incessant arm races described by the so-called Red Queen Hypothesis or antagonistic host–pathogen co-evolution^58^. While the infected host is protected by anatomical barriers and produces antimicrobial proteins and peptides, pathogenic bacteria produce virulence factors, such as proteases to digest host tissues and immune-related polypeptides. Again, as a "counteraction", the host synthesizes and releases proteinase inhibitors to the hemolymph to inhibit the destructive effect of pathogen virulence factors^48^.These properties of GmCP8 allow a conclusion that the protein must have evolved to act not only against intruding bacteria but also against their virulence factors, i.e. proteinases. Additionally, we cannot exclude the possibility that it may also serve another function which is not directly linked to immunity. For example inhibitors of proteases regulate metamorphosis or development, like for example, matrix metalloproteinase inhibitors. Also, when pathogens are encapsulated or nodulated by hemocytes, they can be killed inside the capsule or nodule by released proteases. Then they could be inhibited by compounds secreted by pathogens^59^.

Our results and other findings that GmCP8 is involved in *G. mellonella* immunity were confirmed by the injection of the protein into the larval hemocel 15 min before or one hour after the infection with *P. entomophila*. The most pronounced effect was induced by GmCP8 injected one hour after the injection of *P. entomophila*. It is likely that injected protein persists in the hemolymph for a longer time and then undergoes degradation or inactivation, so we could not observe prolonged survival after the infection with *P. entomophila*.

Overall, the presented results show that, in addition to its opsonin function reported earlier, GmCP8 has antimicrobial activity, which is correlated with changes in the microbial cell surface. Additionally, the protein inhibits the activity of the proteases tested, which leads to a conclusion that GmCP8 is an important component of larval hemolymph participating and maybe regulating defence reactions in *G. mellonella*. Further studies are required to provide comprehensive knowledge of the defence properties of GmCP8.

## Materials and methods

### Insects, microorganisms, immunization, and hemolymph collection

*Galleria mellonella (*Lepidoptera: Pyralidae) were reared on honeybee nest debris at 28 °C and 70% humidity in darkness. Last instar larvae were used for infection.

Bacterial strains used in this study are summarized in Table 1.

**Table 1:** Microorganisms used in the research.

Microorganism	Source/description	Growth conditions	Use in this study
*Pseudomonas entomophila L48*	Frederic Boccard, CNRS, France	LB, 30 °C	Oral and intrahemocelic infection of *G. mellonella; *tests of antibacterial properties of GmCP8
*Pseudomonas aeruginosa*	ATCC 27853	LB, 37 °C	Tests of antibacterial properties of GmCP8
*Escherichia coli D31*	CGSC5165,Genetic Stock Centre, New Haven, CT, USA	LB, 37 °C	Immunization by pricking for preparative purification of GmCP8
*Escherichia coli JM83* carrying plasmid pCH110	Pharmacia-Amersham	LB, 37 °C with ampicillin	Tests of antibacterial properties of GmCP8
*Micrococcus luteus*	ATCC 4698	LB, 37 °C	Immunization by pricking for preparative purification of GmCP8
*Bacillus thuringiensis*	Subsp. kurstaki HD1, Bacillus Genetic Stock Centre, The Ohio State University, Department of Biochemistry	LB, 37 °C	Tests of antibacterial properties of GmCP8
*Staphylococcus aureus*	Collection of the Department of Genetic and Microbiology, UMCS, Lublin, Poland	LB, 37 °C	Tests of antibacterial properties of GmCP8
*Candida albicans*	ATCC 10231	YPD, 37 °C	Tests of antifungal properties of GmCP8

*Pseudomonas entomophila* were grown in liquid medium with 120 rpm rotation (see Table 1 for more details). For oral infection, the overnight culture was sedimented (8500×*g*), washed with Phosphate Buffered Saline (PBS; 140 mM NaCl, 2.68 mM KCl, 10 mM Na_2_HPO_4_, 1.76 mM KH_2_PO_4_ in pH 7.4), and suspended to the density of 10^3^ and 10^5^ colony forming units (CFU) per 10 µl for oral infection by force feeding and 50 cells in 5 µl for hemocelic injection. The number of cells were estimated according to optical density (OD) at 600 nm (for oral infection) and with a cell counter (Muse Cell Analyzer, Especialidades Medicas Myr, S.L., MERCK Millipore) (for intrahemocelic injection), followed by plating the cells and calculation of CFU. As a control, the respective volume of PBS was force fed (10 µl) or injected into the larval hemocel (5 µl).

Immunization with non-pathogenic *E. coli* and *M. luteus* to obtain hemolymph rich in antibacterial compounds was done in the following way. Overnight cultures of bacteria were sedimented (8500×*g*) and the pellet containing live *E. coli* and *M. luteus* was used for immunization by pricking the larvae in the last-but-one proleg with a needle dipped into the pellet. After administration of microorganisms, the larvae were kept in plastic boxes at 28 °C with access to food and good ventilation in darkness.

For hemolymph collection, the larvae were cooled down in ice-cold water. Their body surface was sterilized with 70% ethanol and the larvae were punctured with a sterile needle. The hemolymph was collected to Eppendorf tubes containing a few phenylthiourea crystals to prevent melanization. The hemolymph was then centrifuged at 200×*g* for 5 min at 4 °C to pellet hemocytes, and then at 20,000×*g* for 10 min at 4 °C to get rid of any debris. The collected hemolymph was stored at − 20 °C until use^14,60,61^.

### Preparation of hemolymph for HPLC

Low molecular weight polypeptides were extracted from *G. mellonella* hemolymph as previously described^14,60,61^. In brief, nine volumes of methanol, water, and acetic acid mixture (90:9:1, v:v:v) was added to the hemolymph. Precipitated proteins were pelleted by centrifugation at 20,000×*g* for 30 min at 4 °C. Supernatants containing low-molecular weight proteins were evaporated (Beta 1–8 LD plus vacuum centrifuge, Christ, Germany). Next, the dry deposit was carefully resuspended in 0.1% (v/v) trifluoroacetic acid (TFA) in the volume of two thirds of the initial volume (n) of hemolymph (2/3n). n-Hexane was then added (2/3n) and, after thorough mixing, the samples were centrifuged for 15 min at 4 °C. The upper fraction containing lipids was carefully removed and ethyl acetate of 2/3n was added to the bottom part, mixed, and centrifuged again. The lipid-free bottom part was transferred to new Eppendorf tubes and lyophilized.

### Purification of GmCP8

GmCP8 was purified from freeze-dried and lipid-free *G. mellonella* immunized hemolymph extract prepared as described in the previous section. The extract was dissolved in 0.1% (v/v) TFA and subjected to reversed-phase high-pressure liquid chromatography (RP-HPLC) using a Discovery Bio Wide Pore C18 4.6 mm × 250 mm column (Sigma-Aldrich, St. Louis, MO, USA). The separation was carried out at 40 °C using two buffers, A: 0.1% TFA (v/v) and B: 0.07% TFA containing 80% acetonitrile (both v/v), a linear gradient from 20 to 65% of buffer B in 30 min and a 1 ml/min flow rate. The fraction containing GmCP8 (eluting at 10.5–12.0 min) was collected, freeze dried, and subjected again to chromatography using the same column as above and a linear gradient from 25 to 38% of buffer B in 30 min developed at 20 °C. The GmCP8 fraction (a broad peak eluting at 10.0–12.0 min) was evaporated to dryness and stored at − 20 °C until further assays. The homogenity and identity of purified GmCP8 were confirmed by SDS/PAGE electrophoresis and by N-terminal sequencing performed using an automatic protein sequencer (PPSQ-31A, Shimadzu, Kyoto, Japan). The quantitation of the protein in the solution was determined by the bicinchoninic acid assay (BCA, Sigma-Aldrich, St. Louis, MO, USA).

### In vitro assay of antimicrobial activity of the GmCP8

Overnight cultures of each bacterial species were refreshed by tenfold dilution in LB and grown for additional 2 h at 120 rpm rotation. Twenty microliters of bacteria diluted in a fresh medium to OD_600_ of 0.02 were mixed with the GmCP8 peptide or with water as a control, and then each sample was divided into two parts, 10 µl each. One pair (bacteria with GmCP8 and with water) was incubated for the indicated time (30 min to 120 min) with shaking and then plated, while the second pair was plated immediately (time 0). The final concentration of the GmCP8 peptide mixed with a given microorganism ranged from 3.5 to 14 µM, as indicated in the appropriate Figure.

For plating, control and tested mixtures from the incubation time variants were diluted 100; 1000; and 10,000 times with LB, 900 µl of each dilution was added to LB containing soft (0.7%) agar (w/v) cooled down to 40 °C, and poured on Petri plates. The plates were incubated at 30 °C or 37 °C (depending on the microorganism) until colonies were clearly visible (overnight). The results are presented as a percentage of CFU that appeared after plating the GmCP8-containing samples, in relation to the respective CFU obtained from samples without the peptide^14^.

For testing the activity of GmCP8 against *C. albicans*, logarithmically growing fungi (overnight culture refreshed and grown for 5 h) were used. Fungi at OD_600_ = 0.0025 were mixed with GmCP8 at a final concentration of 7 µM and directly divided into two portions. One was incubated at 37 °C for 60 min, while the other part was plated directly (time 0). For plating, the mixtures (time 0 and 60 min incubation) were diluted 15 and 150 times and 90 µl of each dilution was mixed with YPD containing soft (0.7%) agar and plated on Petri dishes. The number of CFU after mixing the fungi with water (instead of GmCP8) was taken as 100% for the respective time point.

### In vitro membrane permeabilization assay

The membrane permeabilizing activity of GmCP8 was determined on the basis of β-galactosidase leakage from the cytoplasm of *E. coli* strain JM83 carrying plasmid pCH110 (encoding β-galactosidase, Pharmacia-Amersham). The peptide was pre-incubated for 15 min at 37 °C in 20 mM phosphate buffer pH 6.8. Next, 2 µl of a suspension of mild-logarithmic phase *E. coli* JM83 cells (5 × 10^5^ CFU) in the same buffer was added to 23 µl of a GmCP8 solution. The final concentration of GmCP8 was 7 µM or 14 µM. After 45 min incubation at 37 °C, 20 mM HEPES buffer pH 7.5 containing 150 mM NaCl (220 µl) and a 50 mM *p-*nitrophenyl-β-d-galactopyranoside solution (5 µl) were added to the mixture. The samples were incubated for 90 min at 37 °C, and the absorbance at 405 nm was measured. Live bacteria incubated with water in growth medium were used as a negative control (0% perforation), and dead bacteria killed by 5 µM of synthetic cecropin B (Sigma-Aldrich) served as a positive control (100% perforation)^35,62,63^.

### Inhibition of proteases by GmCP8

The GmCP8 peptide at the concentrations indicated in Fig. 5 was mixed with 1 pmol of thermolysin (from *Bacillus thermoproteolyticus rocco,* Sigma) in acetate buffer (10 mM sodium acetate, 5 mM calcium acetate, pH. 7.5) or with 1 pmol of trypsin (from bovine pancreas, Sigma) in reaction buffer: 10 mM Tris–HCl pH 8.0, 0.1 mM HCl. As a control, an equal volume of water was added instead of GmCP8. The blank sample contained the respective buffer instead of the enzyme. EDTA and soybean trypsin inhibitor (STI, Sigma) were used as inhibition control for thermolysin and trypsin, respectively. The samples were pre-incubated for 15 min at room temperature. Then, an equal volume of an azo-casein solution (5 mg/ml in water) was added and the samples were incubated for 1 h at 30 °C . The reaction was stopped by the addition of 10 µl of 5% trichloroacetic acid (TCA), followed by incubation for 10 min at room temperature. Then, the samples were centrifuged at 14,000×*g* for 5 min to get rid of non-digested casein. Nine microliters of 5% NaOH was added to 18 µl of supernatant containing azopeptides released by the enzyme to enhance the colour. The absorbance was measured at 450 nm against a blank sample on clear half-area flat-bottom 96 well plates (Corning) with the use of a Benchmark Plus microplate reader (BioRad). Each assay was performed in at least three replicates.

### Effect of GmCP8 on the survival curves of infected *G. mellonella* larvae

*Galleria mellonella* larvae were injected with 0.1 and 0.2 nmoles of the GmCP8 peptide 15 min before or one hour after infection with 50 cells of *P. entomophila*. The control groups were injected with water, and the insects were reared at 28 °C with access to food. The number of infected living animals was counted at desired time points. The larvae that showed no sign of movement when touched were considered dead. The results are presented with the use of the Kaplan–Meier estimator^64^.

### Isolation of the fat body, RNA extraction, reverse transcription, and qPCR

The larvae were quickly cooled down in ice-cold water and sterilized in 70% ethanol. The fat body was isolated under ice-cold sterile Ringer's solution (172 mM KCl, 68 mM NaCl, 5 mM NaHCO_3_, pH 6.1, osmolarity 420 mOsm; all the ingredients were of cell-culture grade). The organs from five larvae in each group were pulled in ice-cold Ringer's solution into Eppendorf tubes. Then, the liquid was removed and the organs were quickly frozen in liquid nitrogen for at least 10 min and stored at − 80 °C. RNA isolation, reverse transcription (RT), and quantitative PCR (qPCR) were conducted as described before^60,61^. Primers for reference gene S7e and cecropin were described before^33,61^. Primers for GmCP8 were as follows: Forward: 5′-TAGTTGCCGTTCACCGTCTGT-3′, Reverse: 5′-GGGTGTTTCTCCCAGTTCCTT-3′. The results of qPCR were normalised to the level of reference gene S7e, taking into consideration the efficiency of the reaction, which in all cases was above 90%, and shown as relative values with respect to the control samples (obtained from naive larvae)^65^.

### Atomic force microscopy (AFM)

Bacterial or fungal samples were prepared for atomic force microscopy (AFM) according to the method described earlier with minor modifications^35,62^. Briefly, log-phase microorganisms (OD_600_ = 0.02 for *P. entomophila and B. thuringiensis* in LB and OD_600_ = 0.0025 for *C. albicans* in YPD) were cultivated for 1 h at 30 °C with water (control) and with purified GmCP8 (7 µM) in the total volume of 300 µl. Then, 300 µl of 20 mM phosphate buffer, pH 6.8, was added. Next, the pellets were gently washed twice with 20 mM phosphate buffer, pH 6.8, and twice with non-pyrogenic water. After final centrifugation, the microorganisms were suspended in 10 µl of non-pyrogenic water, applied onto the surface of freshly cleaved mica discs, and allowed to dry overnight at 28 °C before imaging. The surface of *P. entomophila, B. thurngiensis*, and *C. albicans* cells prepared on the mica discs was imaged using NanoScope V AFM (Veeco, USA) in the Analytical Laboratory, Faculty of Chemistry, Maria Curie-Skłodowska University, Lublin, Poland. The measurements were carried out in the “Peak Force QMN” operation mode using a silicon tip with a spring constant of 0.4 Nm^−1^ (NSG 30, NT-MDT, Moscow, Russia). The results were processed using Nanoscope Analysis vl. 40 (Veeco). Three fields on each mica disc were imaged. The RMS roughness values and adhesion force values were calculated from 114 fields with an area of 115 nm × 115 nm or 103 nm × 103 nm, respectively, measured over the entire microbial cell surface in areas of 500 nm × 500 nm for *P*. *entomophila* and 1 µm × 1 µm for *B. thuringiensis* and *C. albicans*. The section profiles and three-dimensional (3D) images of the cells were generated using WSxW 5.0 software (Nanotec, Spain)^66^.

### Statistical methods

Statistical analysis was performed using Sigma Plot 12.5 (Systat Software Inc.USA) for all experiments except the membrane permeabilization assay (GraphPad Software Inc.USA) and GLM binomial regression (Microsoft Excel)^67^. Normality of data was assessed with the use of the Shapiro–Wilk test. Significant differences were established at *p* < 0.05. One-way ANOVA followed by post-hoc (Tukey test) tests was used to analyze the results of the antibacterial activity of GmCP8 added at different concentrations against *P. entomophila* and *E. coli* and to analyse the relative density of bands representing the GmCP8 protein, and significant differences were established at p < 0.05. Kruskal–Wallis One-Way ANOVA tests followed by post-hoc test was used to analyse the differences in the gene expression, and significant differences were established at p < 0.05. Values marked with the same letters do not differ significantly. The atomic force microscopy results were analysed using the Mann–Whitney U test. Significant differences are indicated with *p < 0.05, **p < 0.01, ***p < 0.001. In the other experiments, the statistical analysis was performed using the Student’s t-test and significant differences between indicated samples were established at *p < 0.05, **p < 0.01, ***p < 0.001. Welch's t-test was used for determination of differences in membrane permeabilization results, and significant differences were established at p < 0.05. All experiments were performed at least three times.

### Other methods

Tris-Tricine electrophoresis was performed in 16.5%T, 6%C separating gel with “spacer” 10%T, 3%C, and stacking gel 4%T, 3%C^68^. For better visualisation of low-molecular weight proteins and peptides, where indicated, non-stained gels were electroblotted onto PVDF membrane using BioRad equipment, for 1.5 h, 90 V and 200 mA. The membrane was then stained with Coomasie Coomasie Brillant Blue R-250.

## Supplementary Information

Supplementary Information 1.

Supplementary Information 2.

## Data Availability

Any further data (not included in this published article and its supplementary information) that support the findings are available from corresponding author on reasonable request.
