# Supplementary material for: Cationic protein 8 plays multiple roles in Galleria mellonella immunity
Source: Sci Rep. 2022 Jul 11;12:11737. doi: 10.1038/s41598-022-15929-6 (PMC9273619; doi:10.1038/s41598-022-15929-6)
Supplement: Supplementary file 1 — Supplementary Information 1. [file 41598_2022_15929_MOESM1_ESM.pdf]

Cationic protein 8 plays multiple roles in *Galleria mellonella* immunity

Jakub Kordaczuk[1], Michał Sułek[1], Paweł Mak[2], Agnieszka Zdybicka-Barabas[1], Justyna Śmiałek[2] and Iwona Wojda[1]*

[1] *Maria Curie-Sklodowska University, Institute of Biological Sciences, Department of Immunobiology, Lublin, Poland*

[2] *Jagiellonian University, Faculty of Biochemistry, Biophysics and Biotechnology, Department of Analytical Biochemistry, Kraków, Poland*

Supplementary materials: S1, S2, S3 and S6

Supplementary Figure S1

Aligment of aminoacid sequence of Fungal protease inhibitor and Cationic protein 8 precursor.

fungal protease inhibitor-1 [Galleria mellonella] XP_026758048.1

MKAVIILAVL ACVLVATYGD LVCGTNFCKN NPCSTRVAAN SCRSPSVYRQ NHAGKCACCP
ACVTLLPENS ACKTYSKELG ETPSAICREP LKCLNGVCTK IPPRSG

cationic protein 8 precursor [Galleria mellonella] ADI87454.1

MKAVIILAVL ACVLVATYGD LVCGTNFCKN NPCSTRVAAN SCRSPSVYRQ NHAGKCACCP
ACVTLLPENS ACKTYSKELG ETPSAICREP LKCLNGVCTK IPPRSG

Supplementary Figure S2

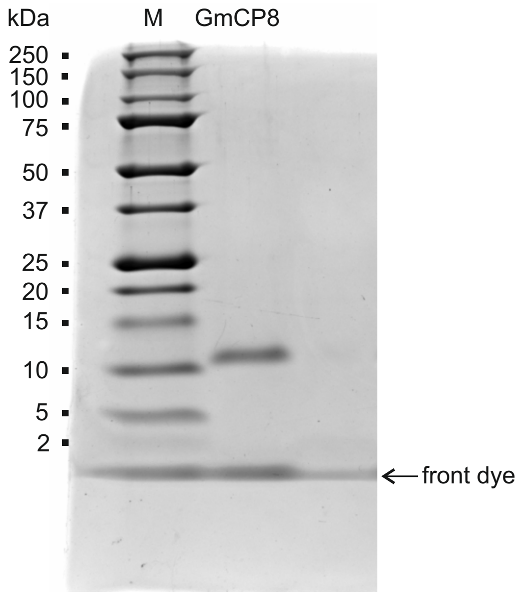

Stained gel after Tris-Tricine electrophoresis
presenting purified GmCP8 protein.
M-molecular weight standards,
The picture of gel was taken
with the use of ChemiDoc instrument (BioRad)

Supplementary Figure S3

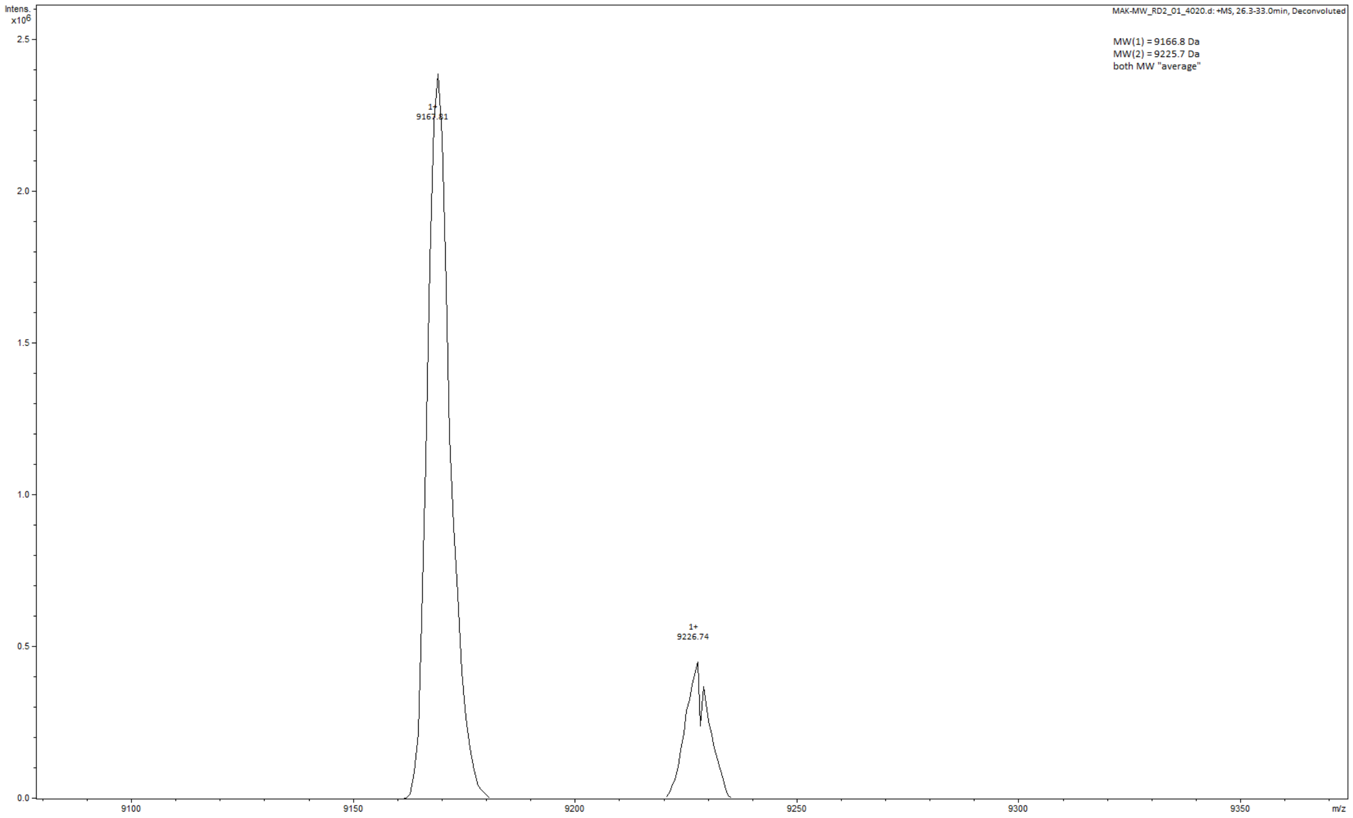

The mass spectrum of isolated GmCP8 protein. The spectrum was measured using an ESI-MS apparatus and subjected to deconvolution to obtain the intact average masses.

Supplementary Figure S6

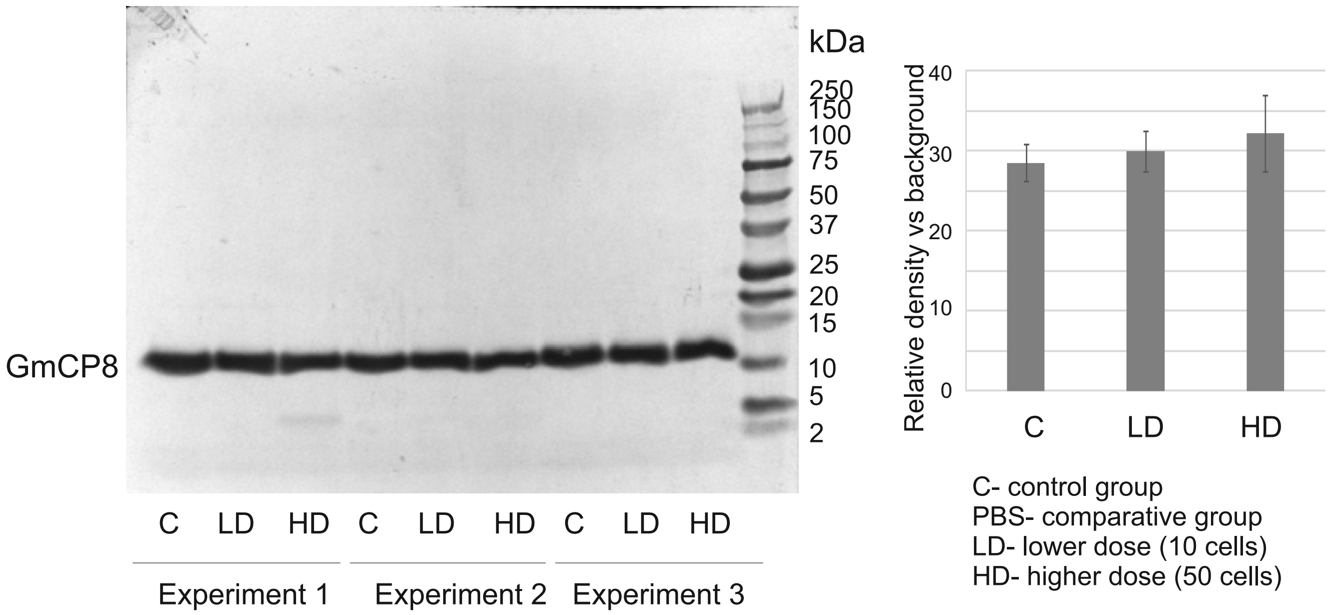

Comparative quantitative analysis of GmCP8 protein present in *G. mellonella* methanol extracts separated by HPLC. **Left**: stained membrane with transferred proteins separated by Tris-Tricine electrophoresis of fraction 20 containing CP8, from control, and infected larvae (injection of *P. entomophila*), from 3 experiments. Identification of protein was done by sequencing N-teminal by Edmann degradation.

Right: relative density band representing GmCP8 protein, from 3 experiments +/- SD.

No significant differences were found (one way ANOVA).
